# Screening and Identification of Molecular Targets Involved in Preventing Gastric Precancerous Lesions in Chronic Atrophic Gastritis by Qilianshupi Decoction

**DOI:** 10.1155/2019/5804710

**Published:** 2019-12-23

**Authors:** Yameng Zhang, Chao Li, Shanmei Sun, Zhiqun Cao, Jian Chen, Hongjie Xiang, Lucheng Song

**Affiliations:** ^1^The First Affiliated Hospital of Shandong First Medical University, Jinan 250355, China; ^2^Shandong University of Traditional Chinese Medicine, Jinan 250355, China; ^3^Digestive Department, Affiliated Hospital of Shandong University of Traditional Chinese Medicine, Jinan 250000, China; ^4^Jinan Central Hospital Affiliated to Shandong University, Jinan 250000, China

## Abstract

Chronic atrophic gastritis (CAG) is a common and possibly precancerous digestive tract disease. Development of drugs with effect of preventing precancerous lesions draws the eyes of global researchers. Qilianshupi decoction (QLSP) is a Traditional Chinese Medicine (TCM) that is commonly used to treat CAG, but few studies have explored the mechanism of QLSP on treating CAG. This study investigated the molecular targets of the component herbs of QLSP in preventing precancerous lesions based on network pharmacology. Network pharmacology analysis revealed that the 6 herbs regulated multiple CAG-related genes, among which the most important were cancer-related pathway (apoptosis, p53, and VEGF) and epithelial cell signaling in *Helicobacter pylori* infection. Further animal experiments showed that the expression of survivin and p53 in precancerous lesions of CAG rats was significantly increased which was suppressed by QLSP. Moreover, telomerase activity was inhibited in precancerous lesions of CAG rats, and telomere length of gastric mucosa was increased, which was reversed by QLSP. Our results suggest that the components of QLSP prevents gastric precancerous lesions through decreasing the expression of survivin and p53 and regulating telomerase activity and telomere length in CAG.

## 1. Introduction

Chronic atrophic gastritis (CAG) is often associated with intestinal metaplasia and atypical hyperplasia, which are characterized by local or extensive chronic inflammation of gastric mucosa, atrophy and reduction of the intrinsic glands, thinning of the mucosal layer and thickening of the mucosal myometrium. CAG is defined as precancerous lesions when accompanied by moderate or severe atypical hyperplasia (ATP) or intestinal metaplasia (IM), according to the 1978 WHO Expert Meeting of Gastroenterology. We must take CAG seriously because gastric cancer (GC) is the fifth most commonly diagnosed malignancy and the third leading cause of cancer-related deaths worldwide [1].

Studies have shown that precancerous lesions are a nonspecific process, characterized by long-term cell degradation and proliferation. Even when some oncogenes are activated, cell metabolism may be deficient, especially nucleic acid metabolism and DNA repair, but these are reversible processes [2, 3]. Precancerous cells can develop to carcinoma *in situ* without interventional therapy or revert to normal cells after treatment.

Moderate and severe atrophic gastritis create a significantly less acidic environment, with decreased parietal cells, G cells, and chief cells in the stomach, which are directly related to decreased hydrochloric acid, pepsinogen, and gastrin [4, 5]. This structural disorder and the decrease in parietal cells diminish the acidic microenvironment and further accelerate malignant transformation. Proliferation and migration of primary cells are also affected by the decrease in parietal cells [6].The decrease of chief cells results in the decreased pepsinogen secretion, and the low-acid environment caused by the loss of parietal cells further decreases the ability to activate pepsinogen, thus creating a vicious circle by aggravating the atrophic gastritis with mucosal nutrition absorption disorder and gland atrophy [7]. The aggravated mucosal atrophy increases gastric ATP and IM, which can lead to gastric cancer. Therefore, preventing further development of IM and dysplasia by CAG is critical to reduce GC incidence, as CAG is an important link in its occurrence and development.

Modern western medicine has no specific treatments for CAG. It is treated by chemotherapy or sequential therapy if *Helicobacter pylori* infection is present; otherwise, it is usually treated with vitacoenzyme [8]. Therefore, choosing an appropriate Traditional Chinese Medicine (TCM) is important. Studies have shown that TCM can reduce or eliminate IM and ATP, thus reversing precancerous lesions and preventing GC [9–12]. Therefore, Qilianshupi decoction (QLSP) was used to treat CAG in our study. QLSP comprises *Astragalus membranaceus*, *Fructus ligustri lucidi*, *Scutellaria barbata*, *Zedoary rhizome*, and *Semen coicis*. We compared the clinical and pathological effects of QLSP and vitacoenzyme for precancerous CAG lesions and found significant differences in clinical symptom score, gastric mirror image, pathological effect, and pathological score (*P* < 0.05) [13]. A randomized controlled trial in rats with precancerous CAG lesions in 2013 showed that QLSP increased gastric mucosal thickness and blood flow and reduced the incidence of CAG precancerous lesions [14].

We repeated the comparison between the two groups and found that QLSP had improved IM pathological score than the vitacoenzyme group. Our experiment showed that QLSP could block or reverse gastric precancerous lesions by such mechanisms as inhibiting angiogenesis factors (such as VEGF and receptor, bFGF, and PDGF), antioxidation, inhibiting lipid peroxidation, regulating immunity, improving blood PGl2/TXA2 balance, inhibiting *H. pylori* infection, and fighting-inflammation [15].

The total effective rate of QLSP in treating CAG in clinical was 80.3%, which was significantly higher than that in the group treated with vitacoenzyme (47.2%). QLSP can notably improve the pathological changes such as granular hyperplasia, hyperemia and edema, and erosion and ulcers in patients who underwent gastroscopy than vitacoenzyme (*P* < 0.01) [16]. The therapeutic effect of QLSP should comprise both direct effects on CAG and indirect effects on other targets. Based on existing pharmacological studies, we found that *Radix astragali* mainly contains polysaccharides, saponins, flavonoids, amino acids, and other chemical constituents [17], which have immunomodulatory, anti-inflammation, antiviral, hepatoprotective, and gastric mucosa protective effects [18]. Extract of *F ligustri lucidi* had an inhibitory effect on tumor cells such as H22, A548, LLC, and LNCap [19]. This antitumor effect was suggested to be caused by inhibition of reverse transcriptase and various DNA polymerases in tumor cells [20]. Methanol extract of *S Coicis* induced apoptosis and cell cycle arrest in human lung cancer A549 cells, *in vitro* and *in vivo*, by reducing mitosis and cell proliferation [21]. Curcumin exerted a significant anti-inflammatory effect in *H. pylori*-infected mucosa, which implies a nutritional approach could prevent *H. pylori*-induced inflammation [22]. Dai used serum pharmacology to study the activity of *S. barbata* against mouse hepatoma H22 cells *in vitro* [23] and found that *S. barbata* extracts could inhibit H22 hepatoma cell growths by inducing apoptosis and cytotoxin.

However, the comprehensive pathway and target of QLSP are still not fully understood. We, therefore, used network pharmacology and bioinformatics to explore the active components and target pathways of QLSP. We screened 74 active components of QLSP and 54 gene targets closely related to the known pathogenesis of CAG. We then screened 132 genes most strongly correlated with QLSP and CAG by analyzing the intersection of drugs and disease targets.

Network pharmacology analysis revealed that the 6 component herbs affected various CAG-related genes, including genes that affect cancer-related pathways (apoptosis, p53, and VEGF), epithelial cell signaling in *H. pylori* infection, TNF signaling, ErbB signaling, toll-like receptor signaling, cAMP angiogenesis, microRNAs in cancer which could cover the main processes of *H. pylori* infection, inflammation, and tumor-like lesions.

In this study, we will detect the expression of survivin and p53, telomerase activity, and telomere length of gastric mucosa cells of experimental rats (as carcinogenesis indicators) to observe the effect of QLSP on CAG gastric mucosal and apoptosis [24, 25]. Therefore, by observing the level of mutant p53 protein expression after QLSP treatment, we will have a clear judgment on the effect of QLSP and the intestinal metaplasia of CAG and observe whether QLSP can inhibit the transformation of CAG to gastric cancer.

## 2. Materials and Methods

### 2.1. Methods of Network Pharmacological Analysis

To study the relationship between QLSP and CAG, we initially screened the known chemical constituents and action targets of QLSP and then screened known targets in CAG pathogenesis. After the intersection was analyzed and predicted, the target was screened again according to the screening rules, and then the possible mechanism was discussed.

We screened the active components of QLSP by searching the Traditional Chinese Medicine Systems Pharmacology Database and Analysis Platform (TCMSP) [26] and Traditional Chinese Medicines Integrated Database (TCMID) [27] and then screened them according to their absorption, distribution, metabolism, and excretion parameters [28–30]. The targets of QLSP were predicted, and the “active component-target network” was constructed by Cytoscape 3.2.1 [31]. Known targets for CAG were retrieved by two databases, OMIM (http://www.omim.org/) [32] and GAD (https://geneticassociationdb.nih.gov/) [33]. We constructed a protein-protein interaction network for treating CAG with QLSP using protein interaction databases. Key targets of CAG by QLSP were screened and analyzed by CytoNCA [34], and mechanisms were analyzed by the gene enrichment analysis plug-in ClueGO [35].

### 2.2. Animals and Drugs

The Experimental Animal Center of Shandong Medical University, Shandong, China (certificate no. SCXK [Lu] 2009–0001) provided Wistar male rats of SPF grade, weighing 62.5 ± 7.5 g. All animal procedures were carried out in accordance with the regulations of the people's Republic of China on the Administration of Laboratory Animals (14 November 1988) and in accordance with the “Guidelines for the Protection and Use of Laboratory Animals” of the National Institutes of Health.

The QLSP drug extract was provided by the pharmaceutical preparation section of Traditional Chinese Medicine Hospital of Shandong Province, at a concentration of 1.2 g/mL. Ranitidine capsule was manufactured by Jiangxi Huiren Pharmaceutical Co., Ltd. N-methyl-N′-nitro-nitrosoguanidine (MNNG) was provided by Germany FluKa products, (batch number: 20090310), Beijing Chemical Reagent Co., Ltd. Ethanol was provided by Tianjin Fuyu Fine Chemical Co., Ltd. (batch no.: 091225). Telo TAGGG Length assay was provided by Roche Co., Ltd.

The MNNG was diluted into 100 *μ*g/mL solution and added to black drinking water bottles. Rats freely drank the freshly-prepared MNNG solution. The MNNG solution was replaced every day for 20 weeks. Ranitidine capsules were added to the diet and processed into a feed containing 0.03% ranitidine and supplemented with irregular diet (full feeding for 2 days and stopping feeding for 1 day recurrently) for 20 weeks. We performed gastric administration of 2 ml 40% ethanol for model rats, twice a week for 10 weeks.

### 2.3. Study Design

**Figure 1:**
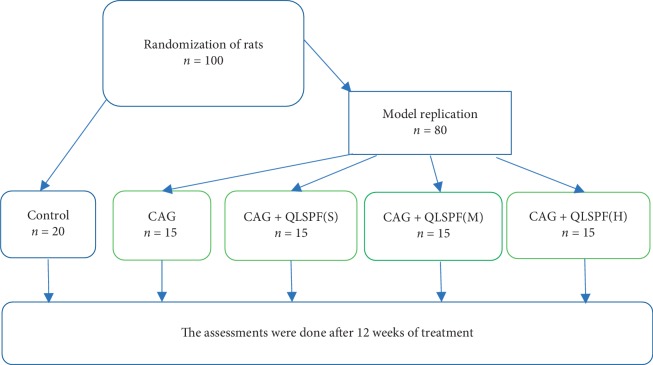
Study design for QLSP treatment of rats with chronic atrophic gastritis.

We randomly divided 100 rats into 2 groups: normal group (*n* = 20), CAG group (*n* = 80). We sacrificed two rats in the CAG group every two weeks to observe the establishment of CAG model until the end of the 20th week. After establishing the model, rats were divided into four groups of 15 rats each according to their body weight: a CAG group, small-dosage QLSP (QLSP(S)) groups, middle-dosage QLSP (QLSP(M)) groups, and large-dosage QLSP (QLSP(L)) groups. Each group received drug interventions for 12 weeks. All groups were given gastric perfusion by body weight at 10 ml/kg. In the QLSP groups, the crude drug content was 0.522 g/mL in the QLSP(L) group, 1.044 g/mL in the QLSP(M) group, and 2.088 g/mL in the QLSP(S) group. The normal and CAG groups were given 0.9% saline.  Figure 1 shows the study design.

### 2.4. Analysis of Telomere Length by Southern Hybridization

Telomere length was measured by Telo TAGGG Telomere Length Assay (Roche). DNA was extracted by the phenol chloroform method and dissolved in TE solution. The integrity of chromosome DNA was detected by electrophoresis. The 20 *μ*l genome was digested overnight at 37°C with an appropriate amount of HinfI, and the degree of digestion was detected by electrophoresis. DNA was digested onto 7 g/L agarose gel for electrophoresis (40 V overnight). The southern blot imprint was then transferred to nylon film. The average telomere length was calculated by hybridization and coloration with kit.

### 2.5. Western Bolt

Cells were lysed with RIPA buffer, and the protein concentration of samples was measured by BCA assay kit (Thermo Scientific). 10–12% SDS-PAGE separating gels were prepared for running protein. After protein transfer, PVDF membranes were blocked in 2% BSA (Sigma-Aldrich) for 1 h and incubated with survivin primary antibody (1 : 1000, abcam #ab469, Cambridge, MA, USA) or p53 primary antibody (1 : 1000, abcam #ab1431, Cambridge, MA, USA) at 4°C overnight and then incubated with the secondary antibody (1 : 20000, Zhongshan Golden Bridge Biotechnology Co, Beijing, China) at room temperature for 1 h. The membranes were infiltrated with ECL (Thermo Scientific) and detected with Immobilon Western HRP substrate (Millipore Corporation, Billerica, USA).

### 2.6. Immunohistochemistry and HE Staining

Tissues were cut into 7 *μ*m slices. After dewaxing, rewatering, and antigen repair, slices were blocked in 4% serum for 1 h and then were incubated with survivin primary antibody (1 : 2000, abcam #ab469, Cambridge, MA, USA) or p53 primary antibody (1 : 100, abcam #ab1431, Cambridge, MA, USA) at 4°C overnight. Slices were incubated with DAB solution, followed by incubation with horseradish peroxidase-labeled secondary antibody for 1 h. The confocal microscope A1 (Nikon Instruments Inc. USA) was used to take pictures. Whether the expression of survivin protein is positive or not depends on the positive integral value of the staining intensity of staining reaction and the number of positive cells. The staining intensity was 0 for nonstaining, 1 for light yellow, 2 for brown yellow, and 3 for brown. The number of positive cells <10% was 0, 10–45% was 1, 46–70% was 2, and >70% was 3. According to the integral value of these two indexes, negative (−) was ≤3 and positive (+) was ≥4. For HE staining, slices were incubated with the alum haematoxylin for 10 min to stain nuclei and stained with eosin for 2 min and then dehydrated, cleared, and measured.

### 2.7. Measurement of Telomerase Activity

Telomerase activity was measured by Telo TAGGG Telomerase PCR ELISA^PLUS^ kit (Roche). Frozen tissue samples were cut into 10 to15 *μ*m thickness, and all steps were performed in accordance with the kit instructions. The absorbance of the samples was recorded by a microplate reader at 450 nm within 30 min after adding the stop reagent.

### 2.8. Statistical Analysis

Data from three independent experiments were expressed as the mean ± standard deviation (SD). One-way ANOVA was used for multiple-group statistical analyses. Raw data were analyzed with SPSS 17.0 software, and images were processed with Graphpad Prism 6. *P* values <0.05 were considered as statistically significant.

## 3. Results

**Figure 2:**
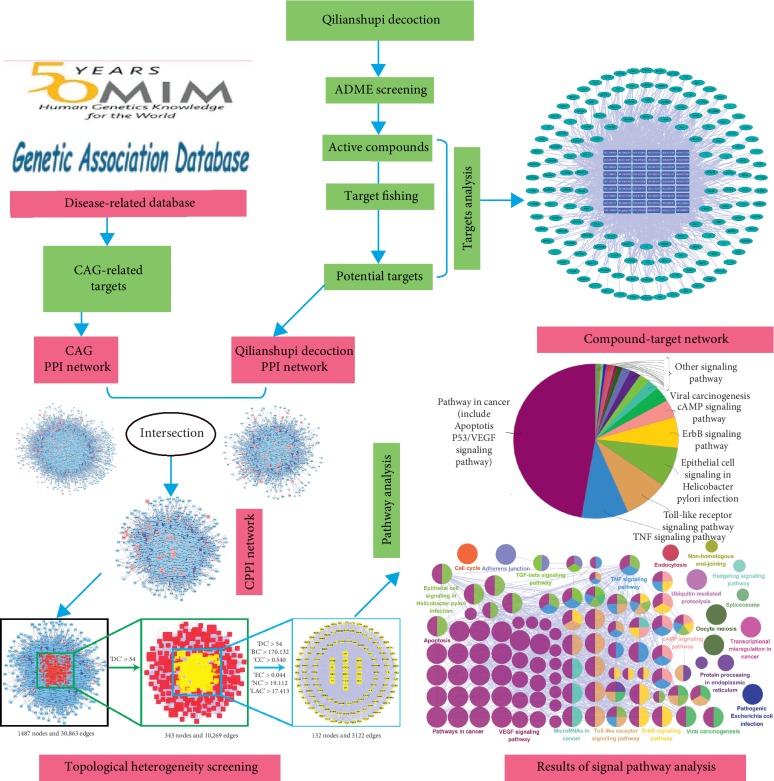
Flowchart of molecular mechanism analysis of QLSP for chronic atrophic gastritis.

Network pharmacology analysis of QLSP with CAG is shown in Figure 2.

### 3.1. Target Prediction and “Active Component-Target Network” Construction

**Figure 3:**
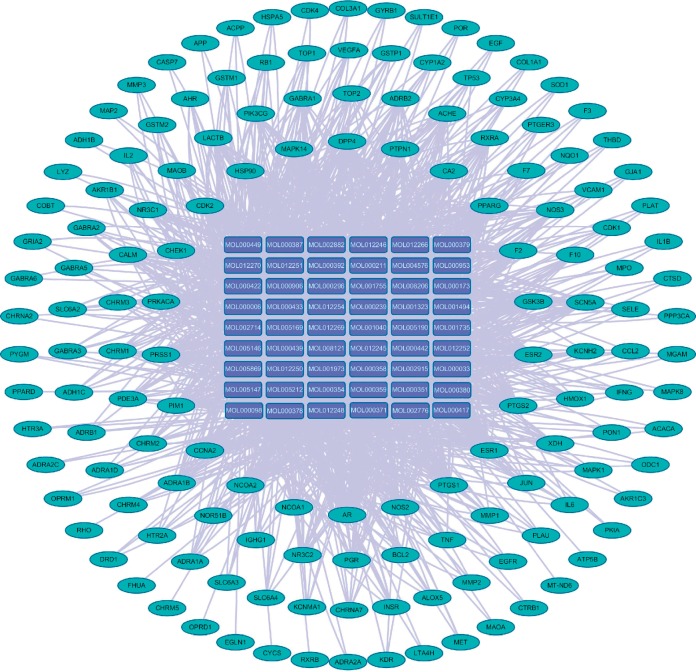
Network chart for targets of active QLSP components.

QLSP is composed of *A membranaceus*, *S. barbata*, *F ligustri lucidi*, *Z rhizome*, *and S coicis*. We searched the TCMSP and TCMID for these components and screened their absorption, distribution, metabolism, and excretion parameters. The screening conditions [36] were oral bioavailability ≥30 and drug-likeness ≥0.18. We obtained 74 active components of QLSP. We predicted the targets of these active ingredients using the TCMSP target prediction model. After excluding repetitive targets, the analysis by Cytoscape 3.2.1 showed 149 active blood-entry components that affect pathogenesis of hematopoietic diseases (Figure 3).

Taking “chronic atrophic gastritis” as the key word, we retrieved only data associated with CAG from the OMIM and GAD databases. We found 54 known target genes for CAG (OMIM: *n* = 35; GAD: *n* = 19).

### 3.2. Screening and Analyzing the Key Nodes of QLSP in Treating CAG

To describe the complex effect of QLSP in treating CAG, we analyzed protein-protein interaction (PPI) data to find direct and indirect regulatory relationships. We consulted four protein interaction relational databases (Biological General Repository for Interaction Datasets, Database of Interacting Proteins, Biomolecular Interaction Network Database, and Molecular Interaction Database) in BisoGenet, to construct the PPI network of the active components of QLSP and CAG. The action pathway of QLSP in treating CAG was a complex regulatory network, and transduction between different signal pathways and targets was clear.

We found 6487 direct or indirect CAG targets of QLSP PPI network and identified as many as 146,962 interactions between these targets. The PPI network diagram of CAG-related targets shows 1984 targets directly or indirectly related to CAG, and 35,960 interactions are related to each other. We therefore extract the intersection network from the QLSP and CAG PPI networks and found 1487 targets and 30,863 interactions by QLSP for the treatment of CAG.

### 3.3. Analysis of the Mechanism of QLSP in Treating CAG

**Figure 4:**
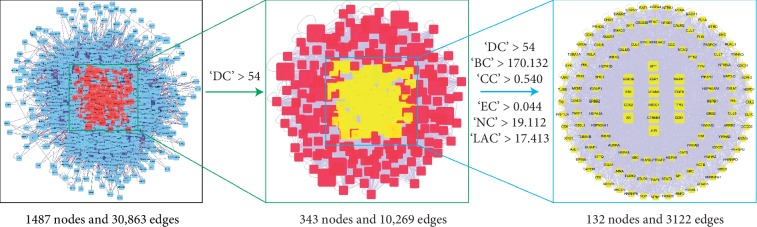
Selection strategy of QLSP for key nodes of chronic atrophic gastritis.

The intersection network extracted from the two PPI network diagrams drawn by Cytoscape, and CytoNCA [34] is used to conduct the network topology analysis by combining the relevant literature and using the indices of degree centrality (DC), betweenness centrality (BC), closeness centrality (CC), eigenvector centrality (EC), network centrality (NC), and local average connectivity (LAC). Initially, we filtered important nodes based on whether the DC value was larger than “big hubs,” which required that the node's DC value be 2× the median DC value of all nodes in the network. We then filtered the new network again, which required the important node to be bigger than the median of DC, BC, CC, EC, NC, and LAC values to ensure that the targets can transmit information through more nodes and have higher information transmission efficiency. We found 132 key nodes by topology screening (Figure 4).

**Figure 5:**
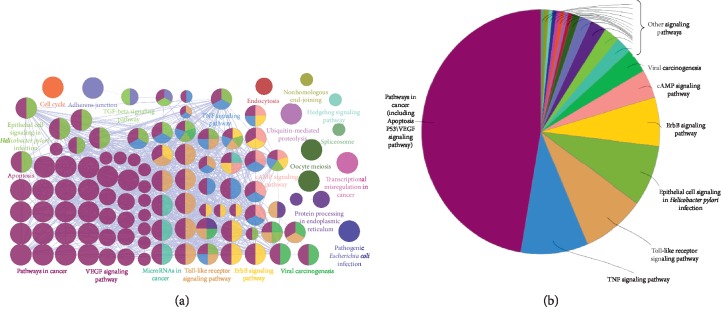
Signal pathway enrichment analysis for the effect of QLSP on chronic atrophic gastritis. Left: most important signal pathway in the group; right: enrichment analysis shown by pie chart.

After these key nodes were found by topology screening, the signal pathway enrichment and KEGG pathway enrichment analysis of 132 key targets were carried out by the gene enrichment analysis plug-in ClueGO, which gave some clues as to the possible mechanism of QLSP in CAG treatment. Network pharmacology analysis revealed that the 6 herbs regulated multiple CAG-related genes, including genes that affect cancer-related pathways (apoptosis, p53, and VEGF), epithelial cell signaling in *H. pylori* infection, TNF signaling, ErbB signaling, toll-like receptor signaling, cAMP angiogenesis, and microRNAs in cancer (Figure 5).

### 3.4. Network Pharmacological Result Analysis and Verification

The most important results are cancer-related pathways (apoptosis, p53, and VEGF), epithelial cell signaling in *H. pylori* infection, and inflammatory pathways. The cancer-related pathways accounts for nearly half of the pie chart area that includes apoptosis, p53, VEGF, MAPK, NF-kappa B, PI3K-Akt, and neurotrophin signaling pathway. Some nodes in Figure 5 named by breast cancer, endometrial cancer, prostate cancer, and thyroid cancer, but further analyzed as MAPK and p53 signaling pathways. Although *H. pylori* infection and inflammation is an essential step in the process of CAG, *H. pylori* is no longer effective once the disease has progressed to IM [37]. IM is a key step in progression towards GC, and how to inhibit IM and regulate cell proliferation and apoptosis is a crucial step in treating CAG [38]. Therefore, it is more important to inhibit the abnormal expression of and apoptosis in the treatment of precancerous lesions of CAG.

The network pharmacological analysis indicated that a significant pathway of QLSP in preventing precancerous lesions of CAG is regulating and apoptosis (Figure 5). In contrast with *H. pylori* infection, the effects of QLSP on and apoptosis of CAG have not been demonstrated, which may be the most critical part of QLSP's suppression of precancerous lesions in CAG.

Therefore, the validation of animal experiments to verify the inhibitory effect of QLSP on *H. pylori* infection is not of particular significance. We chose to study the expression of survivin and p53 and telomerase activity in gastric mucosa of CAG rats to explore whether QLSP inhibits precancerous CAG lesions.

### 3.5. Experiment Results

#### 3.5.1. The Expression of Survivin Was Suppressed in QLSP Groups

**Figure 6:**
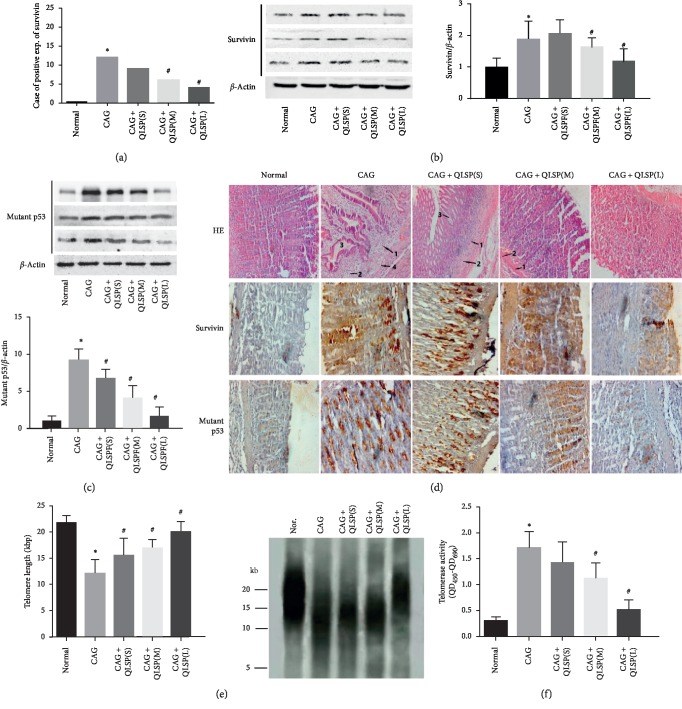
Expression of survivin and mutant p53, histological evaluation of stomach sections, telomere length, and telomerase activity in rat gastric mucosal cells. (a) The positive rate of QLSP in the CAG group was significantly higher than that in the normal group. Survivin expression in the QLSP(L) group was significantly lower than that in the CAG group. (b) Compared with the normal group, expression of survivin in the CAG group was significantly higher than that in the normal group and higher in the QLSP(S) and QLSP(M) groups than that in the QLSP(L) group. (c) Compared with the normal group, the expression of mutant p53 protein was significantly increased in all the groups except the QLSP(L) group. The expression of mutant p53 protein in the QLSP groups was lower than those in the CAG group. (d) Histological evaluation of stomach sections of rats by HE staining. Lymphocytes marked as 1, plasma cells marked as 2, intrinsic glands marked as 3, and lymphatic follicle formation marked as 4. Immunohistochemical photographs of survivin and p53 in gastric mucosa. (e) Telomere length in the CAG group was significantly shorter than that of the normal group and was significantly longer in the QLSP(L) than in the CAG group. (f) Telomerase activity in the CAG group and the QLSP(M) and QLSP(S) group were significantly greater than in the normal group. Telomerase activity in the QLSP(L) group was significantly lower than in the CAG group. ^*∗*^*P* < 0.05 vs. the control group, ^#^*P* < 0.05 vs. the CAG group.

Compared with the normal group, the CAG group had large clusters of brown cells, while the brown cells in the QLSP(L) group decreased significantly than the CAG group in immunohistochemical photographs. The brown cells increased significantly in the QLSP(M) and QLSP(S) group than the QLSP(L) group, but were still less than those in the CAG group (Figure 6(d)). According to the integral value of these two indexes, there was no positive expression of survivin in the normal group but highest in the CAG group. The expression of survivin increased gradually with the decrease of dose in the high- and low-dose group of QLSP (Figure 6(a)). The results suggested that survivin expression in precancerous lesions of CAG rats was significantly increased and that QLSP could dose-dependently inhibit its abnormal expression.

In the gastric mucosa of the CAG group, the expression of survivin gene was significantly increased. But after treatment, the expression of survivin in the QLSP(L) group decreased significantly (Figure 6(b)), which may be one of the mechanisms of QLSP in reversing the precancerous lesions of CAG.

#### 3.5.2. The Expression of Mutant p53 Protein Was Downregulated in QLSP Groups

The brownish red clusters in the CAG group significantly increased than all groups, while there was scarce brownish red clusters in immunohistochemical photographs of the normal group contrarily. The brown cells decreased significantly in the QLSP(M) and QLSP(L) group than the CAG group (Figure 6(d)). We can draw a conclusion that the expression of mutant p53 protein was dose-dependently decreased in all QLSP groups compared with the CAG group (Figure 6(c)), which may be one of the mechanisms of QLSP in reversing the precancerous lesions of CAG.

#### 3.5.3. Histological Evaluation of Stomach Sections of Rats by HE Staining

Compared with the normal group, there are diffuse lymphocytes (Figure 6(d) (1)), plasma cell (Figure 6(d) (2)) infiltration, and lymphatic follicle formation (Figure 6(d) (4)) in the CAG group. At the same time, thinner gastric mucosa and atrophy of intrinsic glands (Figure 6(d) (3)) decreased the number of acid-secreting cell lines, and intestinal metaplasia and dysplasia were observed. There was a dose-dependent improvement in QLSP group compared with the CAG group (Figure 6(d)). The infiltration of lymphocytes and plasma cell decreased significantly in QLSP(M) and QLSP(L) groups. The number of lymphoid follicles in QLSP group was significantly lower than that in the CAG group. The histological evaluation of stomach sections of rats indicated that QLSP can inhibit the transformation of gastric mucosa to precancerous lesions.

#### 3.5.4. The Telomerase Activity Was Decreased in QLSP Groups

From the result of the activity of telomerase quantitatively in gastric mucosa, telomerase activity in CAG group was significantly increased compared with the normal group. Meanwhile, the QLSP(L) group is the closest to the normal group, and the QLSP(M) and QLSP(S) group is closer to the CAG group (Figure 6(f)). Compared with the CAG group, the telomerase activity in the QLSP(L) group was significantly decreased, which indicated that the telomerase activity in precancerous stage of chronic atrophic gastritis was enhanced, and QLSP had a strong inhibitory effect, and the effect was correlated with the dose.

#### 3.5.5. The Telomere Length Shortening Was Inhibited in QLSP Groups

Telomere length in the CAG group and QLSP(M) and QLSP(S) group was significantly shorter than that of the normal group, and the telomere length of QLSP(L) group significantly longer than the CAG group. But the telomere length was longer in QLSP(M) and QLSP(S) groups than in the CAG group (Figure 6(e)). These results suggested that QLSP dose-dependently inhibits telomere shortening in CAG precancerous lesions.

## 4. Discussion

The total effective rate of QLSP in treating CAG in clinical was 80.3%, which was significantly higher than that in the group treated with vitacoenzyme (47.2%). QLSP can notably improve the pathological changes such as granular hyperplasia, hyperemia and edema, and erosion and ulcers in patients with gastroscopy than vitacoenzyme (*P* < 0.01) [16]. The therapeutic effect of QLSP should comprise both direct effects on CAG and indirect effects on other targets. Network pharmacology analysis showed that QLSP should be highly pertinent to CAG treatment in theory, which could cover the main processes of *H. pylori* infection, inflammation, and tumor-like lesions.

The clinical data we collected showed that QLSP can significantly control *H. pylori* infection and inhibit the destruction of gastric mucosal epithelium by *H. pylori*. The eradication rate of *H. pylori* by QLSP was 73.69% [16]. But simply inhibition of *H. pylori* does not reduce the risk of GC in patients with metaplasia and dysplasia. We detected the expression of survivin and p53, telomerase activity, and telomere length of gastric mucosa cells of experimental rats (as carcinogenesis indicators) to observe the effect of QLSP on CAG gastric mucosal and apoptosis [24, 25].

The wild-type p53 gene is a tumor suppressor gene which has biochemical function monitors the integrity of cell genome in G1 phase and prevents the production of cancer-prone mutant cells. However, when p53 gene mutation occurs, it will lose its regulatory function, leading to uncontrolled regulation of cell cycle mediated by p53, disorder of DNA molecular program, genetic instability, and polyploid, leading to malignant proliferation of cells [39]. There was no mutant p53 expression in normal gastric mucosal cells, but the positive rate increased to 60 during the process of chronic atrophic gastritis to intestinal metaplasia and dysplasia [40]. Therefore, by observing the decrease of mutant p53 protein expression after QLSP treatment, we confirmed that QLSP significantly inhibited the intestinal metaplasia of CAG, and we inferred that QLSP could inhibit the transformation of CAG to gastric cancer.

Survivin is a member of the IAPs family of apoptosis suppressor proteins. It is not expressed in normal differentiated tissues but enhanced in most tumor tissues. It mainly inhibits the apoptosis process by inhibiting the caspase apoptotic pathway. Survivin also promotes cell proliferation and angiogenesis. Therefore, its expression may be related to GC carcinogenesis and progression [41–43].

The pathogenicity of survivin expression has three main aspects: (a) it inhibits and disorders apoptosis and stimulates cell proliferation; (b) it stimulates angiogenesis factors and leads to excessive proliferation of vascular endothelial cells, while promoting local ischemia that further activates angiogenesis, thus forming a vicious circle; and (c) its specific binding reaction with spindle microtubules in the premitotic phase causes tumor cells to escape monitoring at the G_2_/M phase of the cell cycle. Resistance to apoptosis induced by DNA damage or mutation may lead to abnormal mitosis and tumor cell proliferation [42–44].

From experimental verification, there was no survivin expression in normal rats but high in CAG rats (Figure 6(a)). The survivin expression in the gastric mucosa of the CAG group was significantly higher than in the normal group (Figure 6(b)). However, survivin expression in the QLSP(L) group decreased significantly after treatment (*P* < 0.01) and decreased to lesser extents in the QLSP(M) and QLSP(S) group (Figure 6(b)), which indicates that QLSP can inhibit survivin expression. We can summarize that QLSP can inhibit the CAG precancerous lesions.

As we know, the pathway from normal cells to cancer cells must involve the mechanism of telomere maintenance, which usually occurs through the upregulation of telomerase. Most somatic cells lack telomerase activity entirely or have very low levels [45]. Both telomerase-dependent and telomerase-independent telomere-elongation mechanisms extend replicative life span and play an important role in cancer progression.

Telomerase is a ribonucleoprotein with reverse transcription activity. Telomere repeat sequences can be synthesized by using the RNA template carried by itself to maintain the stability of telomere length and to make cells divide continuously and become immortalized cells, i.e., malignant tumor cells. Telomere DNA is shortened by 50–100 bp every time a copy is made in normal somatic cells [46], thus acting as a mitotic clock during cell growth and eventually leading to senescence or apoptosis [47]. When normal cells divide, telomeres at the end of the chromosome shorten. When telomerase is activated, telomere length is maintained, and the cell immortalizes [48].

Telomerase activation may link cell immortality and malignant tumorigenesis [49, 50]. Telomerase activity can be detected in 80%–95% of human tumors or tumor-derived cells, but not in normal cells. Telomerase activity showed an increasing trend in normal gastric mucosa during atrophic gastritis and IM dysplasia [51, 52]. Therefore, telomerase activity could be a basis for GC diagnosis and treatment.

Studies suggested that survivin enhances telomerase activity via upregulation of specificity protein 1- and c-Myc-mediated human telomerase reverse transcriptase gene transcription and upregulated telomerase activity by augmenting gene transcription of human telomerase reverse transcriptase (hTERT) [53]. TERT affects cell growth, as it regulates the expression of telomerase, which affects telomere length, and the length of telomeres can affect the cell growth state [54, 55]. Meanwhile, telomerase served as a modulator of NF-kappa B signaling pathway and can regulate the expression of Bcl-2 and survivin downstream [56].

Survivin enhances telomerase activity via upregulation of human telomerase reverse transcriptase gene transcription [53], while immortalization of nontransformed human fibroblasts transduced with telomerase reverse transcriptase upregulates survivin expression [57]. Studies also show that survivin expression and telomerase activity are synchronous expression in cervical cancer and its precursor lesions, and the level of expression of survivin was significantly correlated with the level of human telomerase reverse transcriptase expression and size of the colorectal adenocarcinomas [58, 59].

Wild-type p53 is a powerful inhibitor of human telomerase reverse transcriptase (hTERT), which was one key component for telomerase, but mutant p53 have no similar inhibition [60]. Telomerase activity and protein expression was significantly decreased in H460 (p53 wild-type) cells compared with H1299 (p53 null) cells and p53 knockdown H460 cells (H460-p53-) [61]. These findings suggest that wild-type p53 repressed telomerase activity through downregulation of hTERT transcription, while mutant p53 do not [62–65]. Previous reports suggest that wild-type p53 downregulates the expression of survivin in some cell models and cancer cell lines [66, 67]; meanwhile, the survivin expression is associated with accumulation of mutant p53 in gastric cancer and esophageal cancer [68, 69]. Although there is no literature that mutant p53 can promote the expression of telomerase, a positive correlation between the expression of mutant p53 and telomerase was shown in our study.

The shortening of telomeres may result in chromosome instability and thus promote tumorigenesis. But at the same time, there are studies suggested that telomere shortening in gastric mucosa reflects a field effect in an early stage of carcinogenesis and is associated with an increased risk of GC [70], which is similar to the results in our study. Telomere length in 35 gastric cancers was found shortened significantly compared with the corresponding gastric mucosae in clinical study [71]. “The Singapore Chinese Health Study” demonstrated a higher risk of gastric cancer to be associated with either extremely short or extremely long telomere length. Short and long telomere length may function differently in the early and late stages of gastric carcinogenesis [72]. Increasing evidence has highlighted activities of telomerase that are independent of its conventional function in telomere maintenance which included regulation of cell cycle and gene expression [73–76], inhibition of apoptosis [77], and modulation of cellular signaling such as NF-*κ*B [78]. There are studies showing that the responsiveness and the time needed for effect of telomerase inhibition on growth arrest or cell death would be different based on initial telomere length of target cells, which may lead to repeated changes in telomere length during the gradual formation of gastric cancer. These findings suggest that either short or extreme long telomeres may be a risk factor for gastric cancer [79].

In this experiment, telomerase in gastric mucosa was activated in 15 rats in the CAG group, but was inhibited after QLSP treatment (Figure 6(d)), which implies that QLSP might reverse precancerous lesions by inhibiting telomerase activity.

In spite of telomerase positive expression can inhibit telomere shortening, the length of telomere in IM cells is still shorter than that in normal cells. But when the cell changed from precancerous lesion to gastric cancer, the telomere length in the cell increased significantly [80]. Therefore, the telomere length significantly shortened in precancerous lesions because its telomerase activity may not be strong enough to inhibit telomere shortening or prolong telomere length.

The longest telomere length was in the normal group, followed by the QLSP(L) group. Compared with the normal group, telomere length in the CAG group was significantly shorter than in the normal group, and the telomere length of the QLSP(L) group was significantly greater than in the CAG group (Figure 6(c)). Referring to the inhibition of QLSP on telomerase and surviving and p53, we therefore inferred that QLSP inhibits telomere shortening in CAG precancerous lesions and turned precancerous lesion to normal but GC.

In conclusion, from the point of view of network pharmacology, survivin and telomerase, telomere length measurement, we can conclude that QLSP can effectively inhibit the pathological process of CAG precancerous lesions. And, the abnormal and apoptosis can be adjusted effectively, which has a great significance for the treatment of CAG precancerous lesions.

## Data Availability

All authors allow researchers to verify the results of an article, replicate the analysis, and conduct secondary analyses.

## References

[1] Crew K., Neugut A. I. (2004). Epidemiology of upper gastrointestinal malignancies. *Seminars in Oncology*.

[2] Zhu F. S., Si J. M. (2005). Regulative effect of traditional Chinese medicine on gene-expression related to precancerous lesion of gastric cancer. *Chinese Journal of Integrative Medicine*.

[3] Brower V. (2001). Endostatin “cell factories” shrink rodent brain tumors. *Trends in Molecular Medicine*.

[4] Lacy E. R., Cowart K. S., King J. S., DelValle J., Smolka A. J. (1996). Epithelial response of the rat gastric mucosa to chronic superficial injury. *Yale Journal of Biology and Medicine*.

[5] Genta R. M. (1998). Atrophy and atrophic gastritis: one step beyond the Sydney system. *Italian Journal of Gastroenterology and Hepatology*.

[6] Karam S. M. (1998). Cell lineage relationship in the stomach of normal and genetically manipulated mice. *Brazilian Journal of Medical and Biological Research*.

[7] Broutet N., Plebani M., Sakarovitch C., Sipponen P., Mégraud F., Eurohepygast Study Group (2003). Pepsinogen A, pepsinogen C, and gastrin as markers of atrophic chronic gastritis in European dyspeptics. *British Journal of Cancer*.

[8] Peng L., Xie y.-f., Wang C.-g. (2017). Moxibustion alleviates gastric precancerous lesions in rats by promoting cell apoptosis and inhibiting proliferation-related oncogenes. *African Journal of Traditional, Complementary and Alternative Medicines*.

[9] Wu Y. N., Chen Y. B., Wang W. F., Tu Z. (2005). Clinical study on effects of Kangwei Granule on precancerous lesion in patients with chronic atrophic gastritis. *Zhongguo Zhongxiyi Jiehe Zazhi*.

[10] Yin G. Y., Chen Y., Shen X. J., He X. F., Zhang W. N. (2005). Study on the pathophysiologic basis of classification of ‘spleen’ deficiency in chronic gastritis. *Chinese Medical Journal*.

[11] Xia J. (2004). Medicinal herbs used in pairs for treatment of 98 cases of chronic gastritis. *Journal of Traditional Chinese Medicine*.

[12] Yin G. Y., Zhang W. N., Shen X. J., He X. F., Chen Y. (2004). Study on the pathological basis of classification of spleen deficiency in chronic gastritis. *Chinese Medical Journal*.

[13] Zhao Z. C. H. X. H. (2010). Clinical research of Qilian Shupi granule in the treatment of chronic atrophic gastritis with intestinal metaplasia. *World Journal of Integrated Traditional and Western Medicine*.

[14] Cao H. X. H. W. Z. (2013). Experimental research of the impacts of Qilian Shupi formula on gastric mucosal blood flows and pathological morphology for CAG rats. *World Journal of Integrated Traditional and Western Medicine*.

[15] Cao Z. (2002). *The Study of Qilian Shupi Granule in Treating Precancerous Lesions of Chronic Atrophic Gastritis in Clinic and Inhibiting Factors*.

[16] Yu D. (2003). *The Clinical Study of Treating Precancerous Lesions of Chronic Atrophic Gastritis with Qilian ShuPi Granule*.

[17] Ma X. Q., Shi Q., Duan J. A., Dong T. T. X., Tsim K. W. K. (2002). Chemical analysis of Radix Astragali (Huangqi) in China: a comparison with its adulterants and seasonal variations. *Journal of Agricultural and Food Chemistry*.

[18] OuYang Y., Huang J., OuYang Z., Kang J. (2014). Enrichment and purification process of astragalosides and their anti-human gastric cancer MKN-74 cell proliferation effect. *African Health Sciences*.

[19] Shoemaker M., Hamilton B., Dairkee S. H., Cohen I., Campbell M. J. (2005). In vitro anticancer activity of twelve Chinese medicinal herbs. *Phytotherapy Research*.

[20] Ono K., Nakane H., Meng Z.-M., Ose Y., Sakai Y., Mizuno M. (1989). Differential inhibitory effects of various herb extracts on the activities of reverse transcriptase and various deoxyribonucleic acid (DNA) polymerases. *Chemical & Pharmaceutical Bulletin*.

[21] Chang H.-C., Huang Y.-C., Hung W.-C. (2003). Antiproliferative and chemopreventive effects of adlay seed on lung cancer in vitro and in vivo. *Journal of Agricultural and Food Chemistry*.

[22] Santos A., Lopes T., Oleastro M. (2015). Curcumin inhibits gastric inflammation induced by *Helicobacter pylori* infection in a mouse model. *Nutrients*.

[23] Dai Z.-J., Wang X.-J., Li Z.-F. (2008). Scutellaria barbate extract induces apoptosis of hepatoma H22 cells via the mitochondrial pathway involving caspase-3. *World Journal of Gastroenterology*.

[24] Maruyama Y., Hanai H., Fujita M., Kaneko E. (1997). Telomere length and telomerase activity in carcinogenesis of the stomach. *Japanese Journal of Clinical Oncology*.

[25] Tahara H., Kuniyasu H., Yokozaki H. (1995). Telomerase activity in preneoplastic and neoplastic gastric and colorectal lesions. *Clinical Cancer Research*.

[26] Li Y., Zhang J., Zhang L. (2015). Systems pharmacology to decipher the combinational anti-migraine effects of Tianshu formula. *Journal of Ethnopharmacology*.

[27] Huang L., Xie D., Yu Y. (2018). TCMID 2.0: a comprehensive resource for TCM. *Nucleic Acids Research*.

[28] Liu H., Wang J., Zhou W., Wang Y., Yang L. (2013). Systems approaches and polypharmacology for drug discovery from herbal medicines: an example using licorice. *Journal of Ethnopharmacology*.

[29] Ma C., Wang L., Xie X.-Q. (2011). GPU accelerated chemical similarity calculation for compound library comparison. *Journal of Chemical Information and Modeling*.

[30] Xu X., Zhang W., Huang C. (2012). A novel chemometric method for the prediction of human oral bioavailability. *International Journal of Molecular Sciences*.

[31] Shannon P., Markiel A., Ozier O. (2003). Cytoscape: a software environment for integrated models of biomolecular interaction networks. *Genome Research*.

[32] Hamosh A., Scott A. F., Amberger J. S., Bocchini C. A., McKusick V. A. (2005). Online Mendelian Inheritance in Man (OMIM), a knowledgebase of human genes and genetic disorders. *Nucleic Acids Research*.

[33] Becker K. G., Barnes K. C., Bright T. J., Wang S. A. (2004). The genetic association database. *Nature Genetics*.

[34] Tang Y., Li M., Wang J., Pan Y., Wu F.-X. (2015). CytoNCA: a cytoscape plugin for centrality analysis and evaluation of protein interaction networks. *Biosystems*.

[35] Bindea G., Mlecnik B., Hackl H. (2009). ClueGO: a Cytoscape plug-in to decipher functionally grouped gene ontology and pathway annotation networks. *Bioinformatics*.

[36] Li J., Zhao P., Li Y., Tian Y., Wang Y. (2015). Systems pharmacology-based dissection of mechanisms of Chinese medicinal formula Bufei Yishen as an effective treatment for chronic obstructive pulmonary disease. *Scientific Reports*.

[37] Massarrat S., Haj-Sheykholeslami A., Mohamadkhani A. (2012). Precancerous conditions after *H. pylori* eradication: a randomized double blind study in first degree relatives of gastric cancer patients. *Archives of Iranian medicine*.

[38] Diaz P., Valenzuela Valderrama M., Bravo J., Quest A. F. G. (2018). *Helicobacter pylori* and gastric cancer: adaptive cellular mechanisms involved in disease progression. *Frontiers in Microbiology*.

[39] Kaelin W. G. (1999). The emerging p53 gene family. *JNCI Journal of the National Cancer Institute*.

[40] Shiao Y. H., Rugge M., Correa P., Lehmann H. P., Scheer W. D. (1994). p53 alteration in gastric precancerous lesions. *American Journal of Pathology*.

[41] Tu S. P., Jiang X. H., Lin M. C. (2003). Suppression of survivin expression inhibits in vivo tumorigenicity and angiogenesis in gastric cancer. *Cancer Research*.

[42] Zangemeister-Wittke U., Simon H. U. (2004). An IAP in action: the multiple roles of survivin in differentiation, immunity and malignancy. *Cell Cycle*.

[43] Cai Z., Bao H. Y., Lin M. F. (2005). Correlation between survivin mRNA expression and homoharringtonine induced apoptosis of malignant hematopoietic cells. *Chinese Medical Journal*.

[44] Sanna M. G., Correia J. d. S., Ducrey O. (2002). IAP suppression of apoptosis involves distinct mechanisms: the TAK1/JNK1 signaling cascade and caspase inhibition. *Molecular and Cellular Biology*.

[45] Masutomi K., Yu E. Y., Khurts S. (2003). Telomerase maintains telomere structure in normal human cells. *Cell*.

[46] Griffith J. D., Comeau L., Rosenfield S. (1999). Mammalian telomeres end in a large duplex loop. *Cell*.

[47] Fouladi B., Sabatier L., Miller D., Pottier G., Murnane J. P. (2000). The relationship between spontaneous telomere loss and chromosome instability in a human tumor cell line. *Neoplasia*.

[48] Amioka T., Kitadai Y., Hiyama T. (2004). Expression of human telomerase reverse transcriptase mRNA in esophageal cancers and precancerous lesions. *Oncology Reports*.

[49] Kosciolek B. A., Kalantidis K., Tabler M., Rowley P. T. (2003). Inhibition of telomerase activity in human cancer cells by RNA interference. *Molecular Cancer Therapeutics*.

[50] Newbold R. F. (2002). The significance of telomerase activation and cellular immortalization in human cancer. *Mutagenesis*.

[51] Wong S. C., Yu H., So J. B. (2006). Detection of telomerase activity in gastric lavage fluid: a novel method to detect gastric cancer. *Journal of Surgical Research*.

[52] Nowak J., Januszkiewicz D., Lewandowski K. (2003). Activity and expression of human telomerase in normal and malignant cells in gastric and colon cancer patients. *European Journal of Gastroenterology & Hepatology*.

[53] Endoh T., Tsuji N., Asanuma K., Yagihashi A., Watanabe N. (2005). Survivin enhances telomerase activity via up-regulation of specificity protein 1- and c-Myc-mediated human telomerase reverse transcriptase gene transcription. *Experimental Cell Research*.

[54] Folini M., Brambilla C., Villa R. (2005). Antisense oligonucleotide-mediated inhibition of hTERT, but not hTERC, induces rapid cell growth decline and apoptosis in the absence of telomere shortening in human prostate cancer cells. *European Journal of Cancer*.

[55] Blackburn E. H., Greider C. W., Szostak J. W. (2006). Telomeres and telomerase: the path from maize, Tetrahymena and yeast to human cancer and aging. *Nature Medicine*.

[56] Fatemi A., Safa M., Kazemi A. (2015). MST-312 induces G2/M cell cycle arrest and apoptosis in APL cells through inhibition of telomerase activity and suppression of NF-kappaB pathway. *Tumor Biology*.

[57] Yuan J., Yang B. M.-P., Zhong Z.-H. (2009). Upregulation of survivin during immortalization of nontransformed human fibroblasts transduced with telomerase reverse transcriptase. *Oncogene*.

[58] Lam A. K.-Y., Saleh S., Smith R. A., Ho Y.-H. (2008). Quantitative analysis of survivin in colorectal adenocarcinoma: increased expression and correlation with telomerase activity. *Human Pathology*.

[59] Barbosa L. C. R., da Silva I. D. C. G., Corrêa J. C., Ribalta J. C. L. (2011). Survivin and telomerase expression in the uterine cervix of women with human papillomavirus-induced lesions. *International Journal of Gynecologic Cancer*.

[60] Xu D., Wang Q., Gruber A. (2000). Downregulation of telomerase reverse transcriptase mRNA expression by wild type p53 in human tumor cells. *Oncogene*.

[61] Chen R.-J., Wu P.-H., Ho C.-T. (2017). P53-dependent downregulation of hTERT protein expression and telomerase activity induces senescence in lung cancer cells as a result of pterostilbene treatment. *Cell Death & Disease*.

[62] Daniel M., Peek G. W., Tollefsbol T. O. (2012). Regulation of the human catalytic subunit of telomerase (hTERT). *Gene*.

[63] Rahman R., Latonen L., Wiman K. G. (2005). hTERT antagonizes p53-induced apoptosis independently of telomerase activity. *Oncogene*.

[64] Ruden M., Puri N. (2013). Novel anticancer therapeutics targeting telomerase. *Cancer Treatment Reviews*.

[65] Roake C. M., Artandi S. E. (2017). Control of cellular aging, tissue function, and cancer by p53 downstream of telomeres. *Cold Spring Harbor Perspectives in Medicine*.

[66] Ahn J., Murphy M., Kratowicz S., Wang A., Levine A. J., George D. L. (1999). Down-regulation of the stathmin/Op18 and FKBP25 genes following p53 induction. *Oncogene*.

[67] Yun J., Chae H.-D., Choy H. E. (1999). p53 negatively regulates cdc2 transcription via the CCAAT-binding NF-Y transcription factor. *Journal of Biological Chemistry*.

[68] Yang X., Xiong G., Chen X. (2009). Survivin expression in esophageal cancer: correlation with p53 mutations and promoter polymorphism. *Diseases of the Esophagus*.

[69] Sarela A. I., Verbeke C. S., Ramsdale J., Davies C. L., Markham A. F., Guillou P. J. (2002). Expression of survivin, a novel inhibitor of apoptosis and cell cycle regulatory protein, in pancreatic adenocarcinoma. *British Journal of Cancer*.

[70] Tahara T., Shibata T., Kawamura T. (2016). Telomere length shortening in gastric mucosa is a field effect associated with increased risk of gastric cancer. *Virchows Archiv*.

[71] Yoon J. H., Seo H. S., Choi W. S. (2014). Gastrokine 1 induces senescence and apoptosis through regulating telomere length in gastric cancer. *Oncotarget*.

[72] Wang Z., Koh W.-P., Jin A., Wang R., Yuan J.-M. (2018). Telomere length and risk of developing gastric adenocarcinoma: the Singapore Chinese Health Study. *Gastric Cancer*.

[73] Martínez P., Blasco M. A. (2011). Telomeric and extra-telomeric roles for telomerase and the telomere-binding proteins. *Nature Reviews Cancer*.

[74] Chang S., DePinho R. A. (2002). Telomerase extracurricular activities. *Proceedings of the National Academy of Sciences*.

[75] Smith L. L., Coller H. A., Roberts J. M. (2003). Telomerase modulates expression of growth-controlling genes and enhances cell proliferation. *Nature Cell Biology*.

[76] Sharma G. G., Gupta A., Wang H. (2003). hTERT associates with human telomeres and enhances genomic stability and DNA repair. *Oncogene*.

[77] Lee J., Sung Y. H., Cheong C. (2008). TERT promotes cellular and organismal survival independently of telomerase activity. *Oncogene*.

[78] Park J.-I., Venteicher A. S., Hong J. Y. (2009). Telomerase modulates Wnt signalling by association with target gene chromatin. *Nature*.

[79] Du J., Zhu X., Xie C. (2015). Telomere length, genetic variants and gastric cancer risk in a Chinese population. *Carcinogenesis*.

[80] Maruyama Y., Hanai H., Kaneko E. (1998). Telomere length and telomerase activity in intestinal metaplasia, adenoma and well differentiated adenocarcinoma of the stomach. *Nihon Rinsho*.

